# Bile Duct Targeting or Preservation: Contrasting Liver Histology in Langerhans Cell Histiocytosis and Disseminated Juvenile Xanthogranuloma

**DOI:** 10.1177/10935266251385405

**Published:** 2025-10-31

**Authors:** Margaux Däniker, Frédéric Baleydier, Nathalie M. Rock, Sébastien Menzinger, Barbara E. Wildhaber, Valérie A. McLin, Anne-Laure Rougemont

**Affiliations:** 1 Faculty of Medicine, University of Geneva, Switzerland; 2 Unit of Paediatric Oncology and Haematology, Department of Women, Child and Adolescent, Geneva University Hospitals, CANSEARCH Research Platform in Paediatric Oncology, Switzerland; 3 Swiss Pediatric Liver Center, Department of Women, Child and Adolescent, Geneva University Hospitals, Switzerland; 4 Pediatric Gastroenterology, Hepatology and Nutrition Unit, Division of Pediatric Specialties, Department of Pediatrics, Gynecology, and Obstetrics, Geneva University Hospitals, Switzerland; 5 Division of Clinical Pathology, Diagnostic Department, and Division of Dermatology and Venereology, Department of Medicine, Geneva University Hospitals, Switzerland; 6 Division of Child and Adolescent Surgery, Department of Pediatrics, Gynecology, and Obstetrics, Geneva University Hospitals, Switzerland; 7 Pediatric and Fetoplacental Unit & Clinical Molecular Pathology Unit, Division of Clinical Pathology, Diagnostic Department, Geneva University Hospitals, Switzerland

**Keywords:** secondary sclerosing cholangitis, BRAF, PTPN11, CSF1R, granulomatous hepatitis

## Abstract

Liver involvement by histiocytic and dendritic cell neoplasms signals high-risk disease, often necessitating closer monitoring and aggressive management. Severe cases may progress to liver failure, requiring transplantation. Liver involvement occurs in about one-third of patients with systemic juvenile xanthogranuloma (JXG) and 20% to 60% of pediatric patients with Langerhans cell histiocytosis (LCH), particularly in multiorgan disease. Tyrosine kinase inhibitors show promise in LCH treatment, but optimal timing for treatment cessation remains uncertain. We present 2 pediatric cases, 1 with LCH, and the other with disseminated JXG, along with a literature review emphasizing liver histopathology and transplant considerations. These cases highlight distinct histological patterns. In LCH, progressive bile duct destruction led to ductopenic cholestatic cirrhosis and secondary sclerosing cholangitis. In contrast, in the case of JXG, bile ducts remained intact despite being surrounded by histiocytes. In both, disease localization to larger, segmental portal tracts may reduce liver biopsy sensitivity. In LCH, BRAF inhibitor therapy triggered a granulomatous reaction that could mimic disease recurrence in the liver graft. Other histiocytoses typically spare the bile ducts and do not cause biliary cirrhosis. Recognizing these distinct infiltration patterns can aid diagnosis and management.

## Background

Histiocytic and dendritic cell neoplasms are rare, complex disorders with variable and often non-specific symptoms, depending on whether disease is localized or systemic.

Systemic forms fall under the “L (Langerhans) group” per the 2016 revised classification by the Histiocyte Society.^1^ This group includes Langerhans cell histiocytosis (LCH), Erdheim-Chester disease (ECD), and extracutaneous and disseminated juvenile xanthogranuloma (JXG), all marked by frequent activating mutations in genes of the MAPK pathway, resulting in ERK overexpression.^2^ The 5th edition of the WHO classification of hematolymphoid tumors^3^ divides these neoplasms into 3 categories: (1) Plasmacytoid dendritic cell neoplasms, (2) Langerhans cell and other dendritic cell neoplasms, and (3) Histiocyte/macrophage neoplasms. The latter category groups ECD and JXG together with Rosai-Dorfman disease, ALK-positive histiocytosis, and histiocytic sarcoma. Diagnosis often relies on immunophenotyping, though overlapping features can complicate classification.

Liver involvement occurs in approximately one-third (31.4%) of patients with systemic JXG^4^ and in 20% to 60% of pediatric LCH patients^5^, especially in multiorgan disease, sometimes requiring liver transplantation (LT).

We present 2 pediatric cases, 1 with LCH, and the other with disseminated JXG, highlighting distinct hepatic histology. We also review literature on liver pathology and molecular features in histiocytoses, and the potential role of LT.

## Methods

We reviewed 2 pediatric cases of histiocytosis with liver involvement requiring transplantation. Written informed consent was obtained from the parents. As this study fell outside the scope of Swiss legislation on human research, ethics committee approval was waived.

### Histology, Immunohistochemistry, and In Situ Hybridization

The liver biopsies, explants, and skin biopsies from both patients were processed following standard protocol. For routine histology, 3-μm sections were stained with hematoxylin-eosin (H&E). Representative liver samples were additionally stained with Masson’s trichrome, reticulin, Perl’s, and PAS-diastase. Immunohistochemistry was performed on 4-μm sections formalin-fixed, paraffin-embedded (FFPE) sections using the Ultraview or Optiview detection systems (Roche) for the following antibodies: BRAF V600E, CD1a, CD163, CD68, CK7, Factor XIIIa, Fascin, CD207/Langerin, and S100 protein. Epstein-Barr virus detection was assessed by in situ hybridization using EBER probes. Antibodies and probes are detailed in Table 1.

**Table 1.** Reagents for Immunohistochemistry and In Situ Hybridization.

Antibody	Supplier and clone	Dilution	Detection kit
ALK1	Roche ALK01	RTU	Optiview DAB
BRAF V600E	Roche VE600	RTU	Optiview DAB
CD1A	Leica NCL-L-CD1a-235	1/30	UltraView DAB
CD163	Leica NCL-L-CD163	1/100	UltraView DAB
CD4	Cell Marque 104R-16	1/50	UltraView DAB
CD68	Dako M0876	1/50	UltraView DAB
CK7	Dako M7018	1/100	UltraView DAB
Factor XIIIa	Roche EP3372	RTU	UltraView DAB
Fascin	Dako M3567	1/200	UltraView DAB
Langerin (CD207)	Cell Marque 392M-15	1/50	UltraView DAB
S100	Dako Z0311	1/6000	UltraView DAB
ISH EBER EBV	Roche 800-2842	RTU	Iview Blue

Abbreviations: RTU = ready to use, DAB = 3,3′-diaminobenzidine.

### Molecular Analysis

Tumor cell content was assessed on H&E slides. Genomic DNA was extracted from FFPE tumor tissue using the QIAamp DNA FFPE Tissue Kit (Qiagen). For targeted DNA sequencing, libraries were prepared with a custom 462-gene panel (SureSelect-HS, Agilent) and sequenced on a NextSeq500 (Illumina). Reads were aligned to the hg19 reference genome using BWA-MEM, and variants were called with Mutect2 and Strelka2, using matched normal tissue to filter germline variants.

For RNA sequencing, RNA extracted from FFPE tissue was processed with the TruSight RNA Fusion Panel (Illumina), targeting 507 fusion-associated genes. Libraries were sequenced on a NextSeq, and fusion transcripts were identified using MapSplice 2.1.5.

*BRAF* V600E mutation was excluded by SYBR Green-based qRT-PCR. RNA was isolated (NucleoSpin RNA Kit, Macherey-Nagel), reverse-transcribed (Applied Biosystems, ref. 43698813), and amplified on a LightCycler 480 (Roche), with Ct values normalized to *TBP*.

### Review of the Literature

A systematic search of the MEDLINE (PubMed) database was conducted through June 2025 to identify pediatric cases of liver transplantation due to histiocytic or dendritic cell neoplasms. Search terms included: *pediatric OR children AND liver transplantation AND histiocytosis, OR Langerhans cell, OR juvenile xanthogranulomatosis, OR ALK-positive histiocytosis*.

Liver involvement in LCH was defined by hepatomegaly (>3 cm below the costal margin, midclavicular line) and liver dysfunction (hypoproteinemia <55 g/L and hypoalbuminemia <25 g/L), excluding other causes.^6^ As histological confirmation is not required for diagnosis, we included all reported pediatric liver transplant cases for LCH, JXG, or ALK-positive histiocytosis that provided histological descriptions.

## Results

### Case 1: 5-Year-Old Female at Liver Transplantation—LCH

A 1-year-old female presented with cutaneous lesions diagnosed as LCH), initially managed with topical therapy. Over the following year, she experienced multiple cutaneous flares, which remained responsive to topical treatment. At age 2 years, she developed diabetes insipidus, controlled with desmopressin.

A focal liver lesion was identified during disease work-up and monitored closely. At 2.5 years, she was started on LCH-III protocol treatment (Group 1, multisystem risk). By age 4 years, she developed signs of chronic liver disease, including hepatosplenomegaly, jaundice, and telangiectasias. Liver biopsy revealed sclerosing cholangitis, attributed to LCH.

Soon after, a new skin lesion appeared. Histology showed dermal infiltration by histiocytes with grooved, reniform nuclei and eosinophilic cytoplasm. Cells were positive for CD68, S100, CD207/Langerin, and CD1a. BRAF V600E (VE1) immunostaining was focally positive; mutation analysis confirmed *BRAF* V600E mutation with a variant allele frequency (VAF) of 3.96%, also subsequently detected in blood and bone marrow. Vemurafenib treatment was initiated.

After 4 months, total-body MRI revealed a lytic lesion in the right humerus, suggesting refractory disease. Treatment was discontinued, leading to rapid worsening of cholestasis and necessitating urgent liver transplantation at age 5 years.

The explant liver showed cholestatic cirrhosis with ductopenia, concentric periductal fibrosis around remaining bile ducts, and segmental hypertrophy (segments I and IV) with regenerative macronodules. Cytokeratin 7 highlighted ductopenia, ductular reaction, and aberrant expression in hepatocytes consistent with chronic cholestasis. Langerhans cells (CD68, protein S100, CD207/Langerin, and CD1a positive) were focally present in a large portal tract and gallbladder. NGS confirmed *BRAF* V600E mutation (VAF 0.5%), supporting active liver disease at the time of transplantation, warranting resumption of vemurafenib post-transplant.

The patient has remained on vemurafenib for 8 years post-LT 8 years ago and is disease-free. She undergoes yearly follow up for her LCH including serial liver biopsies. Three years post-LT, protocol biopsies revealed persistent portal and lobular granulomas. However, no recurrence of LCH was seen by immunohistochemistry (S100 and CD1a), and *BRAF* V600E testing has remained negative. Special stains ruled out fungal or mycobacterial infection.

**Figure 1.**
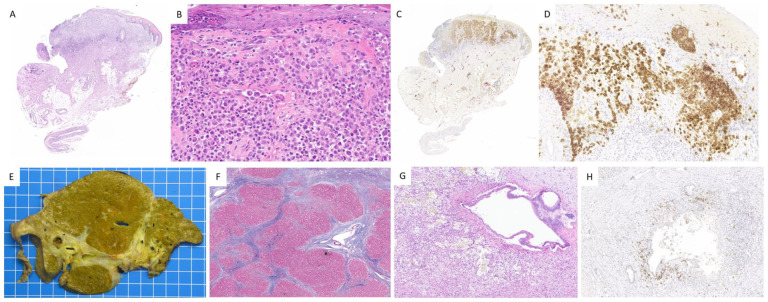
Case 1, LCH. A-D. Skin lesion. The dermis shows infiltration by histiocytes characterized by large vesicular, grooved, and often reniform nucleus, along with moderately abundant eosinophilic cytoplasm (A, B, Hematoxylin and Eosin, H&E). These cells exhibit immunoreactivity for S100 protein (C) and CD1a (D). EH. Liver explant. Cut section shows liver cirrhosis (E), confirmed by Masson’s trichrome (F). A dystrophic, partially disrupted, segmental bile duct is surrounded by a xanthomatous inflammatory reaction (G, H&E), comprising histiocytes reactive to the BRAF V600E (VE1) antibody (H).

**Figure 2.**
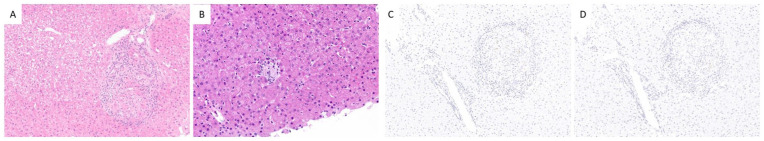
Case 1, LCH. A-D. Vemurafenib-induced granulomatous reaction in the liver transplant. Nonnecrotizing granulomas are seen within both the portal tracts (A) and hepatic lobules (B, H&E). There is no reactivity to S100 protein (C) or to CD1a (D), arguing against recurrent LCH.

Figures 1 and 2 illustrate the main pathological findings and vemurafenib-associated granulomatous changes.

## Main Histological Findings in the Liver: LCH

**Table:** 

• Clinical presentation: cholestasis, liver function preserved. • IHC: CD68+, S100+, and CD1a+. • Location in the liver: infiltration of segmental portal tracts, and of the gallbladder. • Bile duct **tropism** ⇨ ductopenic cholestatic cirrhosis and secondary sclerosing cholangitis.

### Case 2: Infant Male Aged 4 months at Liver Transplantation—Disseminated JXG

A male infant was born at 36 weeks via induction for fetal ascites detected at 33 weeks. Postnatally, he presented with refractory ascites, thrombocytopenia, and an umbilico-iliac porto-systemic shunt on Doppler ultrasound. Portal hypertension raised suspicion for intrahepatic disease, but biopsy was contraindicated due to severe thrombocytopenia.

At 2 months, non-confluent, macular pigmented skin lesions (≤0.4 cm) appeared on the right hemithorax and shoulder, initially attributed to iron overload from transfusions. Despite supportive therapy, he underwent LT at 4 months for cholestatic liver failure and refractory ascites of unknown origin. Pre-transplant liver biopsy was not performed. The post-operative course was mostly uneventful aside from 1 intra-abdominal infection requiring surgical intervention.

At transplantation, hepatomegaly was confirmed (liver weight: 462 g; expected: 160 g). Histology of the explant showed canalicular and hepatocytic cholestasis, bridging necrosis, perisinusoidal fibrosis, and dense histiocytic infiltration of the liver hilum, segmental portal tracts, and round ligament. No Touton cells or xanthomatous giant cells were seen. Infiltrating cells were mononuclear with oval/spindle nuclei, and reactive to histiocytic markers CD68, CD163, and CD4, further expressing Fascin, and Factor XIIIA. They remained negative for CD1a, S100, CD207/Langerin, ALK1, and BRAF V600E; EBER in situ hybridization was negative. Findings were diagnostic of disseminated JXG with liver involvement.

Molecular analysis revealed a class 5 hotspot mutation in *PTPN11* (p.E76V, VAF 34%), and a class 4 *CSF1R* deletion (p.P566_N572del, VAF 20%). Fusion gene screening was negative, including *ALK1* and *NTRK* rearrangements.

Post-LT imaging identified multifocal bone lesions, bilateral renal and pituitary involvement, CNS lesions, and pulmonary infiltration. At 6 months of age, new skin lesions appeared on the face and groin. Biopsies of skin and kidney confirmed histiocytic infiltration with foamy mononuclear cells, sharing the liver immunoprofile, without Touton cells.

Treatment following the multisystem LCH arm of the LCH-IV protocol was initiated at 5 months (1-month post-LT; see Supplemental Figure 1). One year after LT, the patient showed partial systemic remission. Persistent lesions remained in mediastinal vessels, lungs, liver capsule, peritoneum, IVC, meninges, and coronary arteries, but treatment continued without modification.

At 6 years post-LT (and 4 years after completing chemotherapy), the patient remains free of active disease. Imaging shows residual pulmonary and vascular calcifications. Brain MRI at 4 years post-LT revealed white matter rarefaction, periventricular T2 signal changes, and a thin corpus callosum. Clinically, he has normal liver function, few infections, but significant neurocognitive delay and failure to thrive, largely due to oral aversion.

**Figure 3.**
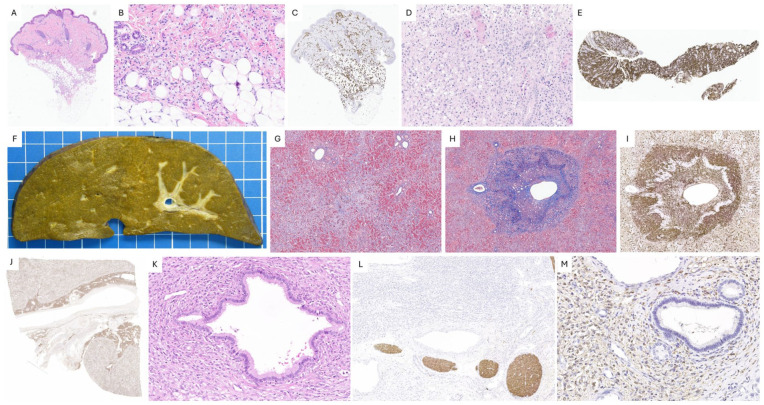
Case 2, JXG. A-C. Skin lesion. The dermis and hypodermis show infiltration by numerous xanthomatous mononuclear foam cells (A, B. H&E), which are positive for CD68 (C). D-E. Kidney infiltration. Renal biopsy reveals extensive infiltration (D, H&E) by similar CD68-positive histiocytes (E). F-M. Liver explant. Nodularity is seen on gross examination (F), while Masson’s trichrome highlights the perisinusoidal fibrosis (G). Centrilobular endotheliitis: histiocytes infiltrate and disrupt the wall of a centrilobular vein (H, Masson’s trichrome, I, Fascin). In the liver hilum, CD68-positive histiocytes form a dense infiltrate (J), surrounding but sparing a large bile duct with intact epithelial lining (K, H&E). These histiocytes are negative for S100 protein, which highlights nerves (L), but show immunoreactivity for Factor XIIIa (M).

Key pathological findings are illustrated in Figure 3.

## Main Histological Findings in the Liver: JXG

**Table:** 

• Clinical presentation: liver function alteration. • IHC: CD68/CD163/CD4+, Fascin+, and Factor XIIIA+. • Location in the liver: >infiltration of the hilum and segmental portal tracts. • Bile duct **preservation** ⇨ histiocytic endotheliitis, bridging necrosis, and perisinusoidal fibrosis.

The main findings from these 2 cases are presented in Table 2. It is noteworthy that both cases were severe and required potentially lifesaving liver transplantation. While they serve as illustrative situations, they do not capture the full spectrum of liver infiltration in LCH and JXG. In particular, JXG often follows an indolent course. Our findings are compared with previously reported histological findings from pediatric liver transplants in LCH and JXG.

**Table 2.** Main Clinical and Histopathological Features of Pediatric Liver Explants in LCH and JXG.

Case #	Age at presentation	Age at LT	Sex	Clinical presentation	Bile duct status	Hallmark cells	Active hepatobiliary disease at LT	Outcome (follow-up)	
**(1) LCH**									
	1 y	6 y	F	Multisystem disease Cholestasis	Sclerosing cholangiopathy Biliary-type cholestatic cirrhosis with ductopenia	S100+, CD1A+, BRAF V600E+ (skin, liver, and gallbladder)	Yes	NED,BRAFi (8y post LT)	*Reported here*
LCH1	19 m	22 m	M	Sclerosing cholangitis with biliary cirrhosis, diabetes insipidus	Cirrhosis, focal LC infiltration	S100+, CD1A+, and Langerin+	Yes	NED (21m)	Wang 2024
LCH2	2.25 ± 2.85	NA, pediatric	NA	Cirrhosis	Pre-transplantation biopsies showing small bile duct sclerosis, “onion-like” changes, LC and inflammatory cells in portal tracts	CD1A+, Langerin+, S100+, and CD68+ (liver biopsy)	No	AWD 4/4 (18-79m) Relapse 18m post-LT (1/4 patient)	Ge 2024
LCH3									
LCH4									
LCH5									
LCH6	2 y	2 y3 m	F	Liver dysfunction	LCH-associated sclerosing cholangitis, cirrhosis	Few CD1A+ cells	Yes	NED (1y)	Watakabe 2023
LCH7	30 m	143 m	M	Multisystem disease Liver dysfunction	Sclerosing cholangitis with ductupenia and cirrhosis	CD1A+ and/or Langerin+ (skin, lymph node, and nail)	No	NED (50m)	Menon 2022
LCH8	8 m	204 m	F					NED (10.5m)	
LCH9	5 m	45 m	F					NED (72m)	
LCH10	30 m	60 m	M					NED (22.5m)	
LCH11	36 m	50 m	F					NED (20m)	
LCH12	3 m	33 m	M					NED (38m)	
LCH13	2 y	2 y1 m	M	Liver dysfunction	LCH-associated sclerosing cholangitis and cirrhosis	CD1A+, Langerin+, and S100+ (liver and extrahepatic bile ducts)	Yes	NED at time of report	Wang 2022
LCH14	1.25 y	6.8 y	NA	Multisystem disease (≥3 organs involved) Sclerosing cholangitis (2/5) Liver dysfunction (2/5) Decompensated cirrhosis (1/5)	Micronodular cirrhosis Sclerosing cholangitis (2/5)	CD1A+ and Langerin+	Yes	AWD (lung, 67m)	Chen 2020
LCH15	1.75 y	4.4 y						NED (38m)	
LCH16	1.25 y	4.3 y						NED (29m)	
LCH17	2.33 y	6.3 y						NED (20m)	
LCH18	1.08 y	2 y						NED (2m)	
LCH19	3 y	3 y3 m	M	End-stage liver disease	Sclerosing cholangitis and cirrhosis, ductopenia, ductular proliferation, cholestasis ; common bile duct: fibrosis, infiltration by LC and eosinophils	S100+, CD1A+, and Langerin+ (extrahepatic common bile duct only)	Yes	NED (4y)	Murakami 2020
LCH20	NA	2 y4 m	M	Multisystem disease Progressive liver disease	Sclerosing cholangitis and biliary cirrhosis	NA	No	NED (2y)	Sheu 2015
LCH21	3 y7 m	4 y	F	Sclerosing cholangitis with biliary cirrhosis	LC surrounding bile ducts, sclerosing cholangitis, and micronodular cirrhosis (ductopenia, ductular proliferation, and cholestasis)	S100+, CD1A+, and CD68+ (liver explant)	Yes	NA	Al Salloom 2013
LCH22	NA	18 m	F	Multisystem disease End-stage liver disease	NA	NA	No	DOC 6m post-LT	Yuksekkaya 2011
LCH23	4 y	6 y	M	Sclerosing cholangitis with biliary cirrhosis	NA	LCH (liver biopsy)	No	NED at time of report	Caruso 2008
LCH24	15 m	3 y	M	End-stage liver disease	Cirrhosis	LCH (skin and liver biopsy)		DOD 1y post-LT	
LCH25									Honda 2005
LCH26	Newborn	2.5 y	F	Biliary cirrhosis	Biliary cirrhosis	CD1A+, Birbeck granules (extrahepatic biliary tree only)	Yes	NA	Jaffe 2004
LCH27	1.5 y	4 y	M	Multisystem disease Cirrhosis	Sclerosing cholangitis and biliary cirrhosis	LCH (skin and liver biopsy)	No	NA	
LCH28	16 m	28 m	F	Cholestasis and malnutrition	Sclerosing cholangitis, ductopenia, and biliary cirrhosis	S100+, CD1A+, Birbeck granules (skin and liver biopsy)	No	NED (16m )	Rajwal 2003
LCH29	NA, pediatric	NA, pediatric	NA	Cholestasis	Sclerosing cholangitis and cirrhosis, large bile duct cystic transformation with bile exteriorization and xanthogranulomatous reaction (2/5)	S100+, CD1A+ in other organs and common bile duct wall (1/5)	Yes (1/5)	2/5 DOC soon after LT	Braier 2002
LCH30									
LCH31									
LCH32									
LCH33									
LCH34	16 m	34 m	F	Multisystem disease Liver dysfunction	Sclerosing cholangitis	S100+, CD1A+ (LCH reactivation in liver transplant)	No	AWD (5y4m)	Hadzic 2000
LCH35	7 m	14 m	F	Severe cholangiopathy	Portal tract histiocytic and neutrophilic infiltration, with bile duct damage and proliferation	S100+ (LCH reactivation in liver transplant); S100+ CD1A+ (skin)		AWD (10m)	
LCH36	NA	6 w	F	Liver dysfunction	Biliary cirrhosis, ductopenia, periductal fibrosis, cholestasis; extramedullary hematopoiesis	S100+, CD1A+ (liver; unclear whether biopsy, explant or autopsy)	NA	NED (5y)	Kaplan 1999
LCH37	NA	1.5 y	F	Hepatosplenomegaly	Fibrosis, bile duct infiltration by LC, ductopenia, ectasia/rupture, periductal fibrosis, and cholestasis			DOD	
LCH38	NA	3 y	F	Multisystem disease	Biliary cirrhosis, bile duct infiltration by LC, ductopenia, ectasia/rupture, periductal fibrosis, and chronic cholestasis			NED (5y)	
LCH39	1 y	3.5 y	NA	Multisystem disease Liver dysfunction	NA	NA	No	2/4 DOC	Newell 1997
LCH40	2.3 y	2.7 y							
LCH41	1.5	4.2 y							
LCH42	1 y	2.4 y							
LCH43	1 y	2.5 y	F	Multisystem disease End-stage liver disease	Sclerosing cholangitis, severe portoportal fibrosis, ductular proliferation, cholestasis ; ulceration of larger bile ducts with xanthogranulomatous reaction	S100+, CD1A+ (skin)	No	AWD (18m)	Melendez 1996
LCH44	0.5 y	14 y	F	End-stage liver disease	Sclerosing cholangitis (1/5) Biliary cirrhosis (5/5) Neoductular proliferation (2/5)	LC in other organs only (skin)	No	NED (8 m)	Zandi 1995
LCH45	3 y	7 y	M			Clinical diagnosis only		Post-operative death	
LCH46	3 y	17 y	F					NED (37 m)	
LCH47	1.1 y	13 y	M			LC in other organs only (skin)		Post-operative death	
LCH48	2 y	12 y	M					NED (9 m)	
LCH49	24 m	5.5 y	M	Multisystem disease, liver dysfunction	Sclerosing cholangitis (1/4), biliary cirrhosis (4/4) with bile duct proliferation	NA	No	NED (4.5 y)	Sommerauer 1994
LCH50	18 m	4.9 y	M					AWD (3.7 y)	
LCH51	26 m	28 m	F					NED (4 y)	
LCH52	18 m	24 m	F					NED (2.4 y)	
LCH53	2 y	2 y3 m	F	Hepatic dysfunction	Sclerosing cholangitis and biliary cirrhosis, infiltration of large bile ducts at the hilum by reactive histiocytes and obliteration, xanthomatous inflammation	Skin biopsy suggestive of LCH, S100+ histiocytes in portal tracts (liver biopsy)	No	NED (30 m)	Squires 1993
LCH54	2.5 y	6.2 y	M	Biliary cirrhosis	Destructive cholangitis (intrahepatic biliary tree), no cirrhosis	Skin biopsy suggestive of LCH	No	NED (34 m)	Rand 1992
LCH55	1 y	1.5 y	F	Multisystem disease Sclerosing cholangitis	Lytic biliary fibrosis, extrusion of bile into the liver parenchyma	LCH diagnosis on skin and gingival lesions		NED (30 m)	
LCH56	20 m	14 y	M	Multisystem disease End-stage liver disease	Liver atrophy, micronodular cirrhosis, massive regenerative nodules, inspissated bile in large bile ducts, calcification and osseous metaplasia, calcified and thrombosed hepatic vein	LC in other organs only (skin and lymph node)	No	NED (2 y)	Mahmoud 1991
LCH57	13 m	3 y	F	End-stage liver disease	Sclerosing cholangitis, destructive cholangitis with focal intraepithelial histiocytes, biliary cirrhosis, xanthomatous lesions with S100+ histiocytes (response to acute cholangitis)?	Skin biopsy suggestive of LCH	No	NED (1 y)	Concepcion 1991
LCH58	5 m	14 y	F	Liver failure	Sclerosing cholangitis and biliary cirrhosis			NED (3 y)	
LCH59	NA, pediatric	NA, pediatric	NA	End-stage liver disease	NA	Diagnosis in referring center	No	NED (5 y)	Stieber 1990
LCH60								NED (4 y9 m)	
**(2) Disseminated JXG**									
	Congenital	5 mo	M	Liver function alteration	Endotheliitis; no bile duct tropism Severe perisinusoidal fibrosis	CD68 +, CD163 +, CD4 +, Fascin +, FactXIIIA + Negative: CD1A, S100, ALK1, and BRAF V600E	Yes	NED (6 y post LT)	*Reported here*
JXG1	NA, pediatric	NA, pediatric	M	Liver failure	Touton giant cells	CD68+, CD163+ (negative: CD1A and S100)	NA	AWD (48.3 m)	Zhao 2024
JXG2	5 y	7 y 8m	F	Liver cirrhosis	Sinusoidal histiocytic infiltration, cirrhosis	CD68 +, CD163 + (negative: CD1A and Langerin)	Yes	Alive, graft enlargment	Irie 2022
JXG3	1y6 m	5y10 m	F	Liver cirrhosis	Sinusoidal histiocytic infiltration, cirrhosis	CD68 +, CD163 + (negative: CD1A, Langerin, and S100)		DOD	
JXG4	2 m	2y2 m	F	Acute liver failure	Sinusoidal histiocytic infiltration, giant cell hepatitis	CD68+, CD163 + (negative: CD1A, Langerin, and S100)		Alive, graft enlargment	
JXG5	2 w	1 m	F	Portal hypertension	Portal tract infiltration	CD68 +, FactXIIIA + (negative: CD1A and S100)	Yes	NED (27 m)	Haughton 2008

Abbreviations: AWD: alive with disease, BRAFi: BRAF inhibitor, DOC: died of other causes, DOD: died of disease, F: female, JXG: juvenile xanthogranuloma, LC: Langerhans cells, LCH: Langerhans cell histiocytosis, LT: liver transplantation, M: male, m: months, NA: non available, NED: no evidence of disease, NOS: not otherwise specified, w: weeks, y: years.

## Discussion

Histiocytic neoplasms range from localized, self-limiting lesions to life-threatening disseminated disease, with potential liver involvement at any stage. Diagnosis is often complicated by overlapping histological features, but infiltration patterns can provide important clues. When involvement is limited to large portal tracts or the hepatic hilum, liver biopsy may be insufficient for accurate diagnosis.

### Liver Involvement by Histiocytic Disorders

Liver involvement in histiocytic disorders can be differentiated histologically by the behavior of histiocytic infiltrates around bile ducts. In LCH, infiltrates cause progressive bile duct destruction, leading to sclerosing cholangitis, whereas in JXG, bile ducts are surrounded but remain intact. Both conditions may ultimately cause biliary-type cirrhosis. Other histiocytoses typically do not target bile ducts or cause biliary cirrhosis.

The clinical presentation and histological patterns of liver involvement in LCH, JXG, and ALK-positive histiocytosis are summarized in Table 3.

**Table 3.** Modes of Presentation and Histological Patterns of Liver Involvement in LCH, JXG, and in ALK-Positive Histiocytosis.

	LCH	JXG	ALK + histiocytosis
Liver involvement in the pediatric population	Hepatobiliary involvement in 60% of multisystemic forms	Rare, <1%	Frequent in multisystemic presentation (17/23, 74%), >infants
Clinical presentation	Hepatomegaly		
	Cholestasis Liver failure End-stage liver disease		Variable non-specific systemic symptoms
Stages/patterns	(1) Proliferative (2) Granulomatous (3) Xanthomatous (4) Fibrosis/Cirrhosis	(1) Early classic (2) Classic (3) Transitional Giant cell hepatitis	Nodular, poorly demarcated Neonatal giant cell hepatitis-like
Cirrhosis	Biliary cirrhosis, cystic changes	Yes, ± perisinusoidal fibrosis	Not reported
Cell morphology	Langerhans cells: round histiocytes with lobulated, central grooving, coffee-bean shaped nucleus, abundant eosinophilic cytoplasm	Mononuclear cells (± foam cells with round to oval nuclei) Few or no giant Touton and non-Touton cells ±Spindle cells	Large histiocytes, with distinctive folded nuclei (may be epithelioid or spindled) Variable foam cells and Touton giant cells May resemble JXG or Erdheim-Chester disease
IHC, positive markers	S100, CD1A, Langerin Histocytic markers (CD68, CD163, and CD4)	Fascin, Factor XIIIA Histocytic markers (CD68, CD163, and CD4)	ALK (membranous and weak cytoplasmic) Histocytic markers (CD68 and CD163) S100 and Factor XIIIA variably Fascin focal
Accompanying infiltrate	Portal eosinophils, neutrophils, lymphocytes (> CD3 +), plasma cells, non-Langerhans histiocytes, multinucleated giant cells	Plasma cells, eosinophils	Many lymphocytes, occasional plasma cells
Portal tracts	Focal infiltration (granuloma-like) Ductular reaction	Granulomas / nodular infiltrates Portal vein infiltration by tumor cells	Large aggregates, ± interstitial fibrosis/sclerosis
Bile ducts and destructive cholangitis	>Small and medium-sized Cholangiocentric Langerhans cell infiltrate Periductal fibrosis → LCH sclerosing cholangitis (15%)	Surrounded but spared → No sclerosing cholangitis	No specific description, presumed to be spared
Lobules	Sinusoidal infiltration Focal, granuloma-like infiltration Confluent tumor-like infiltrates	Granulomas/nodular infiltrates	Single large cells or minute aggregates
Centrilobular vein	Not reported	Infiltration by tumor cells	
Other findings	Cholestasis Macrovacuolar steatosis		Hemophagocytosis
	Macrophage activation	Macrophage activation (Kupffer cell hyperplasia, erythrophagocytosis)	
	Extramedullary hematopoiesis	Hemosiderin deposits	

Abbreviations: JXG: juvenile xanthogranuloma, LC: Langerhans cell histiocytosis.

#### LCH: A Histiocytosis With Selective Bile Duct Affinity

LCH primarily affects children aged 1 to 3 years, with an incidence of 1 to 4 cases per million children annually.^6^ Multisystem involvement is classified as risk organ (RO) positive, or high-risk LCH, when liver, spleen, or bone marrow are affected.^2^ Hepatobiliary involvement occurs in up to 60% of pediatric multisystem cases,^6,7^ whereas isolated liver LCH is rare^8,9^ and often only diagnosed at liver explant histology.^9^ Liver involvement triples mortality risk, accounting for about 20% of deaths in LCH,^2,10^ and cholangitis is associated with significantly worse 2-year overall and progression-free survival.^10^

Clinically, patients may present with jaundice and hypoalbuminemia though, hepatomegaly without cholestasis can reflect macrophage activation syndrome without Langerhans cell infiltration.^11^ Portal tract infiltration by Langerhans cells causes progressive bile duct destruction, advancing from larger to peripheral ducts, leading to sclerosing cholangitis and biliary cirrhosis,^12^ as seen in Case 1. Biliary cirrhosis may develop rapidly within 1 to 3 years, faster than in primary sclerosing cholangitis or that associated with inflammatory bowel disease.^6^ LCH can also cause cystic dilatation of bile duct dilatation due to destructive cholangitis of the larger bile ducts, and present as granulomatous lesions or liver masses.^12^

Four histological stages are recognized: (1) an initial, early proliferative phase, (2) a granulomatous stage, (3) a xanthomatous phase, and (4) a later fibrotic stage.^7,13^ The liver explant from Patient 1 showed late, stage 4 fibrosis with focal xanthomatous activity consistent with stage 3 disease. In the liver, these 4 histological LCH stages correspond to characteristic imaging patterns. During the early stages, periportal inflammation and edema produce bandlike or nodular hypoechogenicity on ultrasound, hypoattenuation on CT, and moderate to high signal intensity on T2-weighted MRI. In the xanthomatous phase, lipid-laden nodules appear hyperechoic on ultrasound, hypoattenuating on CT, and hyperintense on contrast-enhanced T1-weighted MRI without fat suppression. In the later fibrotic stage, typical features of sclerosing cholangitis, such as segmental bile duct narrowing or dilatation with a bead-like appearance, may become evident.^7^ Chemotherapy aimed at inducing remission is most effective during the 3 first stages of active infiltration, whereas the late fibrotic stage typically reflects irreversible disease, with limited treatment options beyond liver transplantation.^14^ Established bile duct injury may persist even if the primary disease becomes “burnt out,” explaining why pretreatment biopsies can be non-diagnostic and liver explants may lack active disease.^11^ Biliary injury can continue to progress despite absence of active disease.^15^

#### JXG: A Histiocytosis Sparing the Bile Ducts

Liver involvement in JXG is exceedingly rare. In a large retrospective series of 525 patients, only 4 cases (<1%) demonstrated hepatic disease, all in individuals under 20 years of age (0.8%).^16^ Similarly, a meta-analysis of 2949 pediatric patients with cutaneous JXG reported systemic manifestations in only 0.75% of cases. Within their own cohort of 338 patients, Samuelov et al. described a 6-week-old infant with multiple hepatic nodules that spontaneously regressed by 7 months of age.^17^ Their literature review identified 10 additional pediatric cases with hepatic involvement,^17-20^ including 1 patient presenting with hepatosplenomegaly but lacking histological confirmation.^19^ Overall, liver infiltration was documented in just 11 of 2949 cases (0.37%).

Notably, a recurrent *NTRK1* fusion has been documented in solitary lesions within the JXG spectrum, giving rise to a provisional new entity of NTRK-histiocytosis.^2,21,22^ This underscores the importance of ongoing molecular refinement in the classification of histiocytic disorders.

#### ALK-Positive Histiocytosis

Liver involvement was first described in 2008 in 3 infants with multisystemic disease.^23^ Patients are classified into 3 groups: Group 1A includes infants with multisystemic disease involving the liver and hematopoietic system; Group 1B comprises patients with multisystemic disease without liver involvement; and Group 2 includes those with single-system disease, where liver involvement is typically absent.^23^ Histologically, the liver shows ALK-positive histiocytic infiltration, with immunohistochemistry enabling the detection of individual infiltrating cells, especially within the lobules.^23,24^ Over time, increasing numbers of Touton and foam cells may appear, potentially mimicking JXG.^24^ Prominent interstitial fibrosis has also been reported, notably in an adult case.^25^

#### Other Histiocytoses and Histiocytic Neoplasms

Indeterminate cell histiocytosis/indeterminate dendritic cell tumor, mainly a cutaneous disorder affecting adults, has rare reports of liver involvement by CD1a-positive histiocytes lacking Birbeck granules ultrastructurally.^26^ This neoplasm is composed of a clonal proliferation of histiocytes expressing the dendritic cells markers CD1a and S100 protein, but not CD207 (Langerin).^3^ Rosai-Dorfman disease^27^ and Erdheim-Chester disease infrequently involve the liver, with the latter rarely diagnosed in children.^28^ Finally, histiocytic sarcoma, a rare malignant histiocytosis involves the liver in approximately 16% of the cases, characterized by sinusoidal infiltration of atypical tumor cells with large, pleomorphic nuclei.^29^

### Molecular Findings and Targeted Therapy, With Focus on LCH and JXG

Histiocytoses are now understood as a spectrum of clonal hematopoietic disorders primarily driven by aberrant activation of the mitogen-activated protein kinase (MAPK) signaling pathway.^21^ Lesions may show mixed phenotypes, combining 2 or more histiocytic disorders, and distinct histiocytoses may co-occur and vary according by anatomical localization.^30^

Recurrent gain-of-function *BRAF*^V600E^ mutations are frequently found in LCH,^31^ while *MAP2K1* mutations typically occur in *BRAF* wild-type lesions, consistent with broad ERK pathway activation in LCH.^30,32^ Other alterations affecting the Ras/Raf/MEK/ERK (MAPK) pathway are identified less frequently in patients lacking *BRAF* or *MAP2K1* alterations. Importantly, LCH cases harboring *BRAF*^V600E^ mutations are linked to higher relapse rates and increased involvement of risk organs such as the liver.^33,34^ Using sensitive techniques like digital droplet PCR (ddPCR), *BRAF*^V600E^ mutations can be detected in liver tissue even without clear histological evidence of active Langerhans cell infiltration, suggesting persistence of clonal mutation-bearing cells in “burnt-out” lesions or circulating precursors”.^34^

This mutation status informs therapeutic approaches, with the BRAF inhibitor vemurafenib demonstrating efficacy in pediatric patients when combined with chemotherapy. However, cessation of vemurafenib often leads to rapid disease relapse,^35^ and sustained remission after treatment cessation remains uncommon, with median remission duration of around 17 months.^36^ The long-term safety and effects of vemurafenib treatment in children still require further evaluation.

In JXG, the MAPK and PI3K pathways are also implicated, though no consistent molecular findings have been identified.^32,37^ Instead of point mutations, *BRAF* fusions are notably enriched in JXG lesions.^38^ Moreover, mutations in the colony stimulating factor-1 receptor (*CSF1R*) gene, which encodes a receptor tyrosine kinase regulating monocyte and macrophage functions, are found in about 10% of JXG cases.^21^ Activation of CSF1R leads to MAPK/ERK pathway signaling.^39^ The p.P566_N572del mutation in *CSF1R*, as observed in Patient 2, affects intracellular domains and results in enhanced kinase activity with cytokine-independent cellular proliferation.^21^ Since CSF-1R signaling is also essential for Langerhans cell development from precursor monocytes,^40^ inhibiting the CSF-1/CSF-1R axis offers a promising therapeutic target for both JXG and LCH.^21,39,40^

The *PTPN11* gene, a key regulator of the RAS/MAPK-pathway, is classically associated with juvenile myelomonocytic leukemia (JMML).^41^ There are documented cases of JXG co-occurring with JMML. For example, 1 male infant diagnosed with JXG at 3 months later developed JMML, and sequencing revealed the same p.E76K *PTPN11* mutation in both diseases.^42^ A hotspot *PTPN11* p.E76V mutation was also detected in Patient 2, who has not exhibited signs of JMML. Furthermore, JXG can occur in the context of Noonan syndrome, a RASopathy caused by germline mutations in genes of the Ras/MAPK pathway, illustrated by a case of a 3-month-old female with a germline p.Y62D *PTPN11* mutation presenting multiple JXG skin lesions.^43^ Coexistence of neurofibromatosis type 1, JXG, and JMML has also been reported.^44^

To our knowledge, co-occurrence of *CSF1R* and *PTPN11* mutations has not been specifically documented in JXG. In Patient 2, the variant allele frequencies were 20% and 34%, respectively, making it unlikely that these alterations arose in separate subclones and were mutually exclusive. This raises the possibility that their simultaneous occurrence exerted a synergistic effect, converging toward more robust and durable downstream MAPK pathway activation, thereby contributing to the increased disease severity observed.

### Indication for Liver Transplantation

In LCH, LT is indicated mainly when disease progresses to sclerosing cholangitis, liver failure, or end-stage liver disease, especially if chemotherapy fails or is not tolerated.^45^ Most LT cases are pediatric (82%), with a median age of 3 years.^9^ At transplantation, over half of patients (56.4%) have active extrahepatic disease, which correlates with a risk of liver recurrence; however, recurrence in the transplanted liver has been reported in only 8% of patients and is generally responsive to salvage therapy.^9^ Thus, LT can be considered in selected patients with decompensated liver disease and active LCH when chemotherapy is ineffective or contraindicated. Maintenance therapy with vemurafenib post-LT, as in Patient 1, has shown durable disease control, although optimal treatment duration remains unclear.

LT has been reported in 59 pediatric LCH patients,^10-15,46-64^ with active liver disease at the time of transplantation in 12 cases,^11,46,49,54,61-63^ including Patient 1. Recent cases show favorable outcomes, with follow-up up to 5.5 years^49,54,61-63^ Notably, the case we report remains disease-free 8 years post-LT while continuing BRAF inhibitor therapy, reflecting an unusually prolonged favorable evolution.

For disseminated JXG, only 6 patients including the case discussed here have undergone LT, with the youngest at 1 month old.^65-67^ Although Patient 2 met LT criteria at age 1 month, transplant was deferred until age 5 months due to risk considerations.

No reports of LT exist for ALK-positive histiocytosis.

### Potential Mimics for Recurrence in Liver Transplants

Pathologists should be aware of 2 potential diagnostic pitfalls when evaluating suspected recurrence: (1) granulomatous reactions (GRs), and (2) presence of CD1a+, Langerin+, Langerans cells in portal tracts in association with cholangiopathy.

(1) Adverse cutaneous events to BRAF inhibitors (BRAFi) occur in more than 75% of the patients, with GRs increasingly recognized. These GRs may resemble multiorgan sarcoidosis-like disease but are more commonly limited to the skin.^68^ GRs occur mainly after dabrafenib (59%) and vemurafenib (37%) use, with 1 reported pediatric case involving skin-only lesions.^69^ A review by Pham et al.^68^ identified 55 BRAFi-related GR cases, with only 1 pediatric patient. Time to onset varies widely (1 month-5 years, mean = 10 months). Liver involvement as granulomatous hepatitis is rare.^70^ GRs affect 4.2% to 6% of histiocytosis patients on BRAFi.^68^ In the context of known histiocytic neoplasms, granulomas may signal recurrence. Patient 1 remains on vemurafenib, currently tolerating treatment despite GR, raising questions about treatment duration and long-term side effects versus recurrence risk.
(2) Entenmann et al.,^71^ reported CD1a+, Langerin+ Langerhans cells infiltrating bile duct epithelium or portal connective tissue during acute cellular rejection (ACR) without evidence of LCH. These Langerhans cells represent an inflammatory response associated with ACR-related cholangiopathy and should not be mistaken for LCH recurrence.

In both scenarios, immunohistochemical and molecular testing for known mutations is essential to avoid misdiagnosis of recurrence, allowing accurate assessment within the graft.

In conclusion, hepatic involvement in histiocytosis may lead to end-stage liver disease requiring transplantation. Histiocytoses display distinct hepatic infiltration patterns that aid diagnosis. Targeted therapies, particularly tyrosine kinase inhibitors, have transformed management, but determining when and whether to discontinue treatment remains a critical and unresolved challenge.

## Supplemental Material

sj-pptx-1-pdp-10.1177_10935266251385405 – Supplemental material for Bile Duct Targeting or Preservation: Contrasting Liver Histology in Langerhans Cell Histiocytosis and Disseminated Juvenile Xanthogranuloma Supplemental material, sj-pptx-1-pdp-10.1177_10935266251385405 for Bile Duct Targeting or Preservation: Contrasting Liver Histology in Langerhans Cell Histiocytosis and Disseminated Juvenile Xanthogranuloma by Margaux Däniker, Frédéric Baleydier, Nathalie M. Rock, Sébastien Menzinger, Barbara E. Wildhaber, Valérie A. McLin and Anne-Laure Rougemont in Pediatric and Developmental Pathology
