# Supplementary material for: Bile Duct Targeting or Preservation: Contrasting Liver Histology in Langerhans Cell Histiocytosis and Disseminated Juvenile Xanthogranuloma
Source: Pediatr Dev Pathol. 2025 Oct 31;29(1):38–50. doi: 10.1177/10935266251385405 (PMC12779774; doi:10.1177/10935266251385405)
Supplement: sj-pptx-1-pdp-10.1177_10935266251385405 – Supplemental material for Bile Duct Targeting or Preservation: Contrasting Liver Histology in Langerhans Cell Histiocytosis and Disseminated Juvenile Xanthogranuloma [file sj-pptx-1-pdp-10.1177_10935266251385405.pptx]

## Slide 1
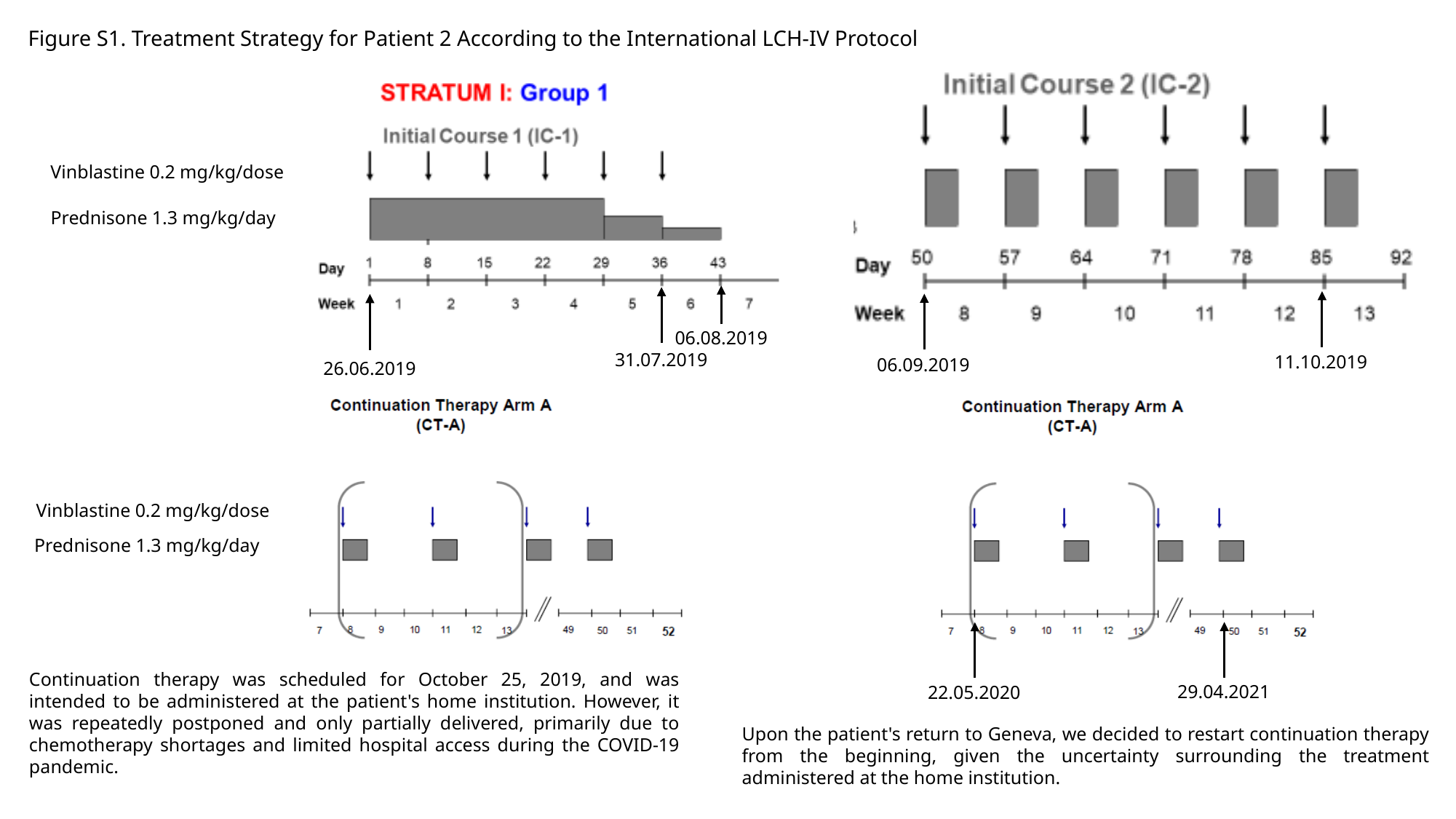

Figure S1. Treatment Strategy for Patient 2 According to the International LCH-IV Protocol
Vinblastine 0.2 mg/kg/dose
Prednisone 1.3 mg/kg/day
06.08.2019
31.07.2019
11.10.2019
06.09.2019
26.06.2019
Vinblastine 0.2 mg/kg/dose
Prednisone 1.3 mg/kg/day
Continuation therapy was scheduled for October 25, 2019, and was intended to be administered at the patient's home institution. However, it was repeatedly postponed and only partially delivered, primarily due to chemotherapy shortages and limited hospital access during the COVID-19 pandemic.
29.04.2021
22.05.2020
Upon the patient's return to Geneva, we decided to restart continuation therapy from the beginning, given the uncertainty surrounding the treatment administered at the home institution.
